# Evolution of transcriptional regulation in closely related bacteria

**DOI:** 10.1186/1471-2148-12-200

**Published:** 2012-10-06

**Authors:** Olga V Tsoy, Mikhail A Pyatnitskiy, Marat D Kazanov, Mikhail S Gelfand

**Affiliations:** 1 Institute for Information Transmission Problems, RAS, Bolshoi Karetny per. 19, Moscow, 127994, Russia; 2 Faculty of Bioengineering and Bioinformatics, Moscow State University, Vorobievy Gory 1-73, Moscow, 119992, Russia; 3 V.N. Orekhovich Institute of Biomedical Chemistry, RAMS, Pogodinskaya St. 10, Moscow, 119121, Russia

## Abstract

**Background:**

The exponential growth of the number of fully sequenced genomes at varying taxonomic closeness allows one to characterize transcriptional regulation using comparative-genomics analysis instead of time-consuming experimental methods. A transcriptional regulatory unit consists of a transcription factor, its binding site and a regulated gene. These units constitute a graph which contains so-called “network motifs”, subgraphs of a given structure. Here we consider genomes of closely related Enterobacteriales and estimate the fraction of conserved network motifs and sites as well as positions under selection in various types of non-coding regions.

**Results:**

Using a newly developed technique, we found that the highest fraction of positions under selection, approximately 50%, was observed in synvergon spacers (between consecutive genes from the same strand), followed by ~45% in divergon spacers (common 5’-regions), and ~10% in convergon spacers (common 3’-regions). The fraction of selected positions in functional regions was higher, 60% in transcription factor-binding sites and ~45% in terminators and promoters. Small, but significant differences were observed between *Escherichia coli* and *Salmonella enterica*. This fraction is similar to the one observed in eukaryotes.

The conservation of binding sites demonstrated some differences between types of regulatory units. In *E. coli*, strains the interactions of the type “local transcriptional factor ➝ gene” turned out to be more conserved in feed-forward loops (FFLs) compared to non-motif interactions. The coherent FFLs tend to be less conserved than the incoherent FFLs. A natural explanation is that the former imply functional redundancy.

**Conclusions:**

A naïve hypothesis that FFL would be highly conserved turned out to be not entirely true: its conservation depends on its status in the transcriptional network and also from its usage. The fraction of positions under selection in intergenic regions of bacterial genomes is roughly similar to that of eukaryotes. Known regulatory sites explain 20±5% of selected positions.

## Background

Currently Genbank contains more than a thousand complete bacterial genomes and many more are in progress [1]. On the other hand, the regulation of gene expression was experimentally studied in detail only for a few model organisms, such as *Escherichia coli*, *Salmonella enterica*, *Bacillus subtilis*, or selected functional systems of particular interest in other species.

However, the availability of numerous genomes at different levels of taxonomic closeness now allows one to use bioinformatic methods relying on statistical analysis and comparative genomics to reconstruct transcriptional regulatory interactions in sets of related species either starting from experimental data such as known regulatory sites or genes changing expression in certain conditions [2-7], or *de novo*[8-14], for reviews see [15,16]. Such analyses can be done for particular transcription factors and regulatory systems (reviewed in [16]) or for entire taxa [17-19], or for transcription-factor families [20-22].

In each cell, transcription factors (TFs), their binding sites and regulated genes form transcriptional regulatory networks (TRNs). Compared to a random graph, natural TRNs contain an excess of so called “network motifs” [23], or “graphlets” [24], that is, subgraphs with a given structure. One of the most abundant motifs is the feed-forward loop (FFL) [25]. A FFL comprises three genes, two TFs and one regulated gene. The first TF controls the second TF and both of them control the third gene.

FFLs can be further classified based on the type of regulatory links or TFs. Each of the three interactions in a FFL can be either activating or repressing [26]. FFL is called coherent if the first TF has the same direct effect on the regulated gene as its indirect effect via the second TF. Otherwise, it is called incoherent [23]. From the biological point of view, incoherent FFLs might be important for the transient response to persistent signals [27]. Moreover, incoherent FFLs can speed up the response time of the network acting as sign-sensitive accelerators, while coherent FFLs act as sign-sensitive delays [26].

**Figure 1:**
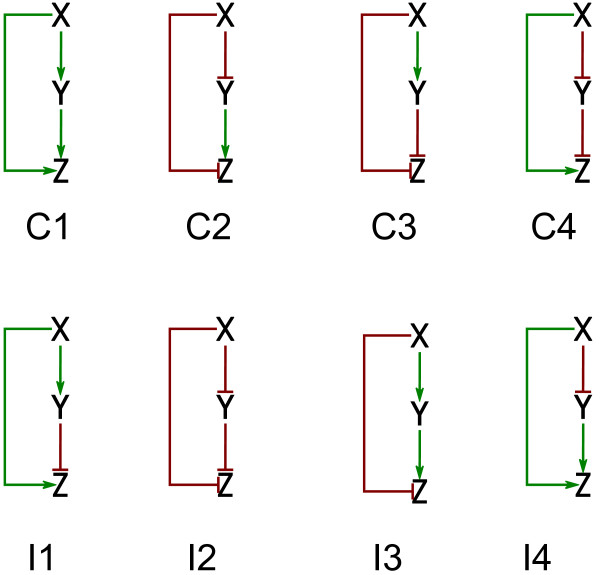
Types of feed-forward loops (FFLs).

Not all FFLs occur equally often. The coherent FFL with three activation interactions is the most common one in *E. coli* (type C1 in Figure 1). In the most frequent incoherent FFL, the first TF upregulates the expression of the second TF and the gene while the second TF downregulates the expression of the gene (type I1 in Figure 1). At that, the differences in the frequencies of the FFL types are not explained simply by the relative abundances of repressor and activator interactions in the network [26]. 

It has been suggested that the TRN evolution depends on the type of the regulator action. Activators are more likely than repressors to be lost when their targets are maintained. In order for a repressor to be removed, its targets need either to acquire alternative regulation, or to be lost themselves. So the repressors with many targets turned out to be more conserved than activators [28].

All regulatory links can be classified depending on whether the TF is global or local. Previous analysis of the network context demonstrated that most FFLs belong to two types [29]. Either both TFs regulate a large number of genes and one of them regulates the other, the archetypal example being FNR and ArcA of *E. coli*[30], or the first TF is a global regulator, and the second TF, a local one, as exemplified in *E. coli* by CRP and local sugar regulators [31]. Three possible definitions of global regulators are feasible. The subsystem-based approach defines global TFs based on their ability to regulate different metabolic pathways [32]. The regulation-based approach uses such criteria as the number of regulated genes, e.g. more than 15 genes [33] or 10 operons [23]. Additional criteria are the number and types of co-regulators, the variety of conditions in which the regulatory interactions are invoked, etc. [34]. Finally, the network-based approach identifies as global those TFs that regulate several modules in the TRN [35]. In the *E. coli* TRN, these approaches are consistent only for seven regulators: CRP, IHF, FNR, Fis, ArcA, Lrp, H-NS. Other candidates are NarL, Fur, Mlc [33], CspA, OmpR, RpoN, RpoS [35]. In this work we combined these approaches to define global regulators.

The abundance of the FFLs yields the question of their evolutionary significance. Previously, the FFL motif conservation has been shown to correlate with the lifestyle defined as a set of several parameters like oxygen requirements, optimal growth temperature, environmental condition and pathogenicity, so that organisms that share a similar lifestyle tend to conserve similar transcriptional regulatory network motifs [27]. Also, the conservation of regulatory links that form FFLs was claimed to be more correlated than the conservation of triples in random regulatory interactions or pairs of co-regulated genes [36]. However, in both these studies an interaction was considered to persist simply when the TF and the regulated gene were present, whereas orthologous TFs in bacteria may have different functions and regulate different genes [37]. Here, we analyzed not only the conservation of TFs and regulated genes, but also the conservation of the TF binding sites upstream of the genes, assuming that if the site is conserved, then the regulation is maintained.

The bacterium arguably best studied from the regulatory point of view is *E. coli* K12. We used the available data collected in the RegulonDB database [38] to study evolutionary changes in transcriptional regulation of related species. At that, we compared the behavior of the TRN connections forming FFLs to those not belonging to motifs. We did that at different taxonomy levels: from *E. coli* strains, where we required absolute conservation of a site, to the Enterobacteriales order, where we required existence of a site with the score close to the score of the original site.

A related problem is that of the evolutionary forces acting on regulatory sites and, more generally, intergenic regions. In a series of papers, Lassig and coworkers demonstrated that that even modest positive selection is sufficient to create a TF-binding site in a relatively short time [39,40] and that the calculated strength of TF-binding sites is more conserved than expected given the sequence conservation level [41], demonstrating specific purifying selection on the former. In yeasts, the fraction of positions in intergenic regions subject to purifying selection is ~40% [42], while for *Drosophila simulans* introns it is ~45% [43]. For *E. coli*, an estimate based on comparison to synonymous codon positions is ~50% [44]. The latter study used a complicated statistical procedure to account for the fact that, at least in bacteria, the existence of the codon usage bias demonstrates that synonymous positions are not neutral, and thus cannot serve as a straightforward control.

We propose a simple method for estimating the fraction of positions subject to purifying selection in non-coding DNA regions given two sets of strains of two related species. These species should be sufficiently close so that the intergenic regions could be unambiguously aligned, yet a substantial fraction of nucleotides had been substituted since the species diverged from their common ancestor. We apply this method to the genomes of *E. coli* strains and *Salmonella* spp.

Overall, we utilize a large number of available, completely sequenced genomes at different levels of taxonomic relatedness to characterize the TRN evolution using a variety of newly developed computational methods and comparative approaches. Our aim is to determine how changes in different units such as regulatory sites, regulated genes, transcriptional factors and network motifs contribute to this process.

## Results

### Selection in intergenic regions

We suggest that the fraction of sites evolving under purifying selection can be estimated by comparing conservation statistics of orthologous intergenic regions in alignments from clades of closely related bacterial genomes. The genomes should be sufficiently close in order (1) to allow for unambiguous identification of orthologous genes; (2) to retain a considerable fraction of the gene order so that orthologous intergenic regions could be identified as regions between pairs of pairwise orthologous genes; and (3) to allow for alignment of orthologous intergenic regions.

**Figure 2:**
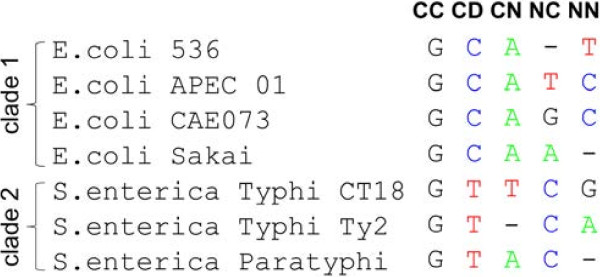
** Five types of alignment positions for sequences from two clades (*E. coli* and *Salmonella* spp.).**

Each alignment position for the two clades may be unambiguously classified as belonging to one of the following types (Figure 2):

(i) conserved and identical in both clades (*CC*);

(ii) conserved in both clades but differs between them (*CD*);

(iii) conserved in the first clade but variable in the second clade (*CN*);

(iv) variable in the first clade but conserved in the second clade (*NC*);

(v) non-conserved in both clades (*NN*).

We assume that an alignment position is evolving neutrally (purifying selection does not act) if it contains at least one substitution. Still, even a neutrally evolving position may contain no substitutions, if the genomes are close: insufficient time may have passed since species shared their common ancestor for a substitution to occur. Let *s* be the total number of neutrally evolving positions. For the neutral positions we may write a contingency table (Table 1). Assuming that functionality of a position does not differ between the clades and the substitution rates are the same in the two clades, we obtain:

(1) $$s=\frac{\left(NN+CN\right)\left(NN+NC\right)}{NN}$$

**Table 1:** Distribution of neutrally evolving positions

	**Variable positions in the** ***Salmonella*** **clade**	**Conserved positions in the** ***Salmonella*** **clade**	**Total**
Variable positions in the *E.coli* clade	*NN*=170±40	*NC*=749±113	*NN*+*NC*
Conserved positions in the *E.coli* clade	*CN*=1370±291	*CD*=3823±422 and an unknown fraction of *СС*=17541±1709	not relevant
Total	*NN*+*CN*	not relevant	*s*

Since the total number of positions in the alignment, *n*, and the numbers of alignment positions of each type (*CC*, *CD*, *CN*, *NC* and *NN*) are directly observable (Table 1), the fraction of positions under purifying selection can be easily calculated as ω=1−s/n. The mean values and standard deviations for all parameters were obtained for 100 bootstrap samples of 15 *E. coli* strains and 15 *Salmonella* strains. In the calculation above only alignment positions without gaps were considered. Assuming that a position containing a gap is neutral, we multiplied ω by the fraction of ungapped alignment positions for each genome.

To assess the robustness of the observed estimates, we performed a two-stage bootstrap procedure with resampling of both genomes and alignment positions. At the first (external) cycle we tested whether the estimated fraction of positions under purifying selection depended on the choice of genomes included in the alignment. We randomly selected 15 *E. coli* strains and 15 *S. enterica* strains, extracted orthologous intergenic regions, aligned them and estimated the fraction of positions under selection as described above. The cycle was performed 100 times. At the second (internal) cycle we tested the dependence on positions in the multiple alignment. For the current set of genomes we constructed a bootstrap alignment, where each column was sampled independently with replacement from the initial alignment. This cycle was performed 10 times.

**Figure 3:**
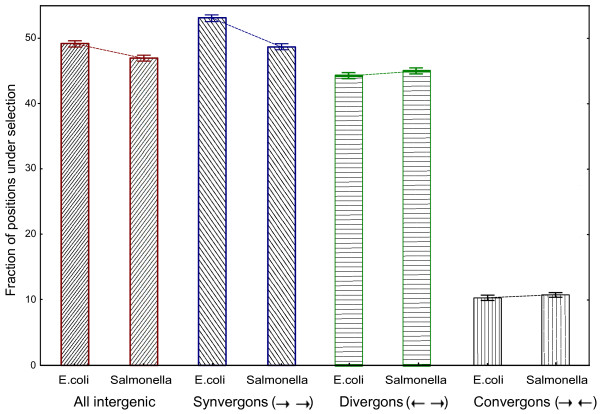
** Fraction of positions under selection for different types of intergenic regions.** Whiskers designate 95%-confidence interval for the mean

For each genome we calculated the mean fraction of non-coding positions under selection, ωω and its 95% confidence interval. The fraction of positions under selection was estimated for all intergenic regions, for convergons (genes sharing 3’-regions), divergons (genes sharing 5’- regions) and synvergons (consecutive genes from the same strand). The obtained estimates are shown in Figure 3.

Unexpectedly, the highest fraction of positions under selection, approximately 50%, was observed in the synvergon intergenic regions, followed by divergons (slightly more than 44%) and convergons (approximately 11%). Small, but significant differences were observed between *E. coli* and *S. enterica* according to the Mann–Whitney test for all intergenic regions and for synvergons (both p-values <0.001). Changes in fractions of positions under selection in convergons for *E. coli* and *S. enterica* were significant (p-value=0.01), while we found no significant differences between divergons (p-value=0.29).

**Figure 4:**
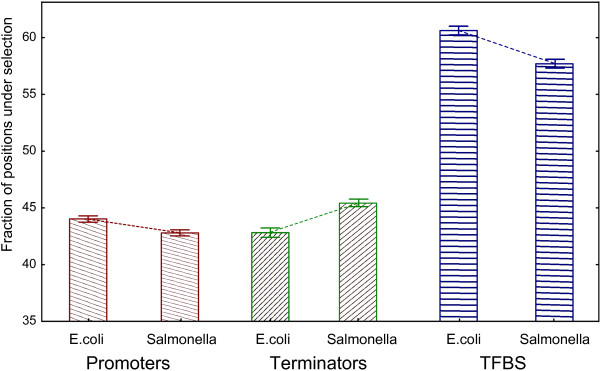
** Fraction of positions under selection for different functional DNA regions.** Whiskers designate the 95%-confidence interval for the mean

We also estimated the fraction of positions under selection in different functional DNA regions of *E. coli* K12, such as promoters, terminators and TF-binding sites (TFBS) according to RegulonDB. The fraction of positions under selection for TF-binding sites was higher than in the intergenic regions in general, (~60%) while values for promoters and terminators were approximately the same (45%) (Figure 4). Again, significant differences were observed between *E. coli* and *S. enterica* according to the Mann–Whitney test in all cases (p-values<0.001).

### Definition of global regulators

As the TRN evolution might depend on the regulator type, it is necessary to define global regulators. We analyzed the number of the regulated operons (Additional file 1: Table S1) and the diversity of the metabolic pathways.

The largest regulons were observed for all seven universally accepted global regulators and FUR. FUR controls several distinct cellular processes: acid and oxidative stresses, glycolysis and gluconeogenesis, phage DNA packaging [45,46], metal ion stress [47-49], resistance to cobalt and nickel [50], the tricarboxylic acid cycle, porins, respiration, purine metabolism, flagellum chemotaxis, methionine biosynthesis [51-53], 2,3-dihydroxybenzoate biosynthesis [54], hence we classified it as a global regulator.

While such TFs as NsrR, LexA, CpxR, NarL have large regulons, we considered them as local, since each of their regulons is involved in a single cellular process. Indeed, NsrR regulates genes involved in cell protection against nitric oxide (NO) [55,56], LexA mediates SOS-response [57], CpxR is involved in conjugation [58], NarL controls anaerobic electron transport and fermentation-related genes in response to availability of high concentrations of nitrate or nitrite [59].

### Conservation of regulatory interactions in *E. coli* strains

A connection in a TRN may change for a variety of reasons: the regulated gene, TF or TFBS might disappear. Also, the evolution of binding sites is not strictly qualitative: a site may be present, but with a changed binding rate. In very closely related species (in our case, the *E. coli* strains), the TRN does not change dramatically, as changes affect TF-binding sites (TFBS) rather than TFs or regulated genes. Thus, analyzing strains we are able to see the contribution of TFBS gains/losses to the TRN evolution.

We analyzed TFBS in 25 *E. coli* strains. This resulted in 355 links involved in FFLs and 367 links in non-motif connections, that is, connections that do not form FFLs. We divided them into 105 non-motif links with global TFs, 262 non-motif links with local TFs, and 194 global TF and 161 local TF links involved in FFL.

All obtained regulatory links from *E. coli* K12 were propagated to other strains. A TFBS was assumed to be non-conserved if it had at least one substitution in any strain. Of 199 global links, 165 were conserved: 63 of non-motif interactions and 102 links from FFLs. Of 423 local links, 261 remained: 152 of non-motif interactions and 109 links from FFLs. The chi-square test showed that the local links were slightly more conserved in FFLs compared to non-motif interactions (p-value 0.047).

**Figure 5:**
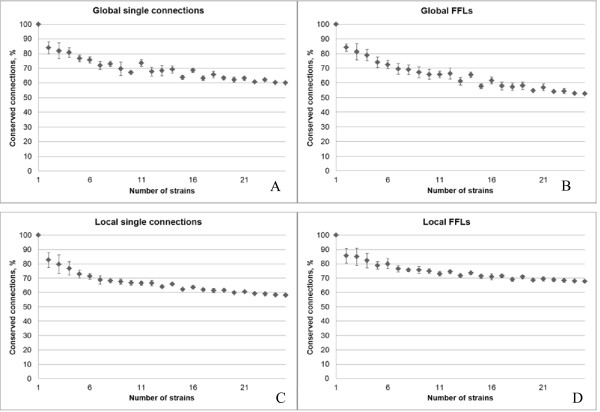
** The fraction of conserved links depending on the number of studied strains.A**. global non-motif links; (**B**) links from global FFLs; (**C**) local non-motif links; (**D**) links from local FFLs

The validity of this analysis depends on the data robustness, namely, whether the results will change dramatically after adding new, distantly related strains. We calculated how the proportion of conserved regulatory links depends on the number of analyzed strains. This proportion stabilized starting at 15 ± 2 strains (Figure 5A, B, C, D).

### Conservation of regulatory interactions in the Enterobacteriales

In the Enterobacteriales, the TRN undergoes all possible events mentioned above: the TF, TFBS or regulated gene gain/loss. As the *E. coli* TRN is the best charcterized one among bacteria, propagating this TRN to the Enterobacteriales by identifying conserved interactions yields a reasonable model of the TRN evolution.

We analyzed only experimentally determined regulatory links from the RegulonDB database [38] and further restricted the analysis to those TFs, for which a PWM could be produced. This yielded 96 TFs. The final sample contained 473 non-motif connections and 418 connections involved in FFLs.

As TFs might be global (G) or local (L), there exist six possible types of regulatory interactions: G➝gene, L➝gene, G➝G, G➝L, L➝L, L➝G, where “gene” means a gene not encoding a TF, that is, a terminal node in the TRN. The case L➝G appeared only once with the pair NsrR➝Lrp. Also, the G➝G interaction was observed only in FFLs. Hence, we analyzed the conservation of FFL and non-motif interactions of four types: G➝L, L➝L, G➝gene, L➝gene.

We analyzed all possible events at a regulatory link. All three elements (TF, TFBS, a gene) might be conserved (referred to as “Conserved links”). Further, the TF might disappear (“No TF”); an orthologous TF might be present, but the regulated gene lost (“No regulated gene”), and, finally, the TFBS might be absent with both TF and gene being conserved (“No TFBS”). In the case of multiple TFBSes for the same TF upstream of the same gene, we considered separately the situation when all binding sites, hence, the entire regulatory interaction, were lost. The number of lost and conserved regulatory links are given in Table 2. The last column represents these numbers specifically for *S. enterica*.

**Table 2:** The number of events in TRN

	**G➝L**	**L➝L**	**G➝gene**	**L➝gene**	**L➝gene (specifically in** ***S. enterica*)**
Non-motif connections					
No TF	0	73	0	240	45
No regulated gene	31	8	607	674	26
No TFBS (at least 1)	7	62	708	1201	14
No TFBS (all)	1	25	596	746	11
Conserved links	51	321	840	2522	250
Total links	89	452	2155	4637	335
FFL					
No TF	0	189	0	208	44
No regulated gene	281	82	572	193	15
No TFBS (at least 1)	186	117	828	395	17
No TFBS (all)	105	44	536	186	8
Conserved links	290	483	1310	596	110
Total links	757	871	2710	1371	186

For all analyzed species, the link L➝gene was slightly more conserved in non-motif connections compared to the FFL ones. The chi-squared p-value for *S. enterica* is 0.006. For other Enterobacteriales, the p-values are not significant, but the same effect is present in all of them.

No significant differences were observed for global regulators, so the conservation of their regulatory links does not depend on participation in the FFL motif.

We further analyzed the most abundant coherent and incoherent types of FFLs: types C1 and I1 respectively (Table 3, Figure 1). The type C1 FFLs tend to be less conserved than the type I1 FFLs. The TFBSs in the C1 FFLs disappear at a faster rate than in the I1 FFLs. This behavior does not depend on the type of the regulated gene.

**Table 3:** The number of events in TRN

	**G➝L**	**L➝L**	**G➝gene**	**L➝gene**
C1 FFLs				
No TF	0	72	0	138
No regulated gene	157	15	131	32
No TFBS (at least 1)	50	40	282	184
No TFBS (all)	10	14	178	77
Conserved links	87	107	483	147
Total links	294	234	896	501
I1 FFLs				
No TF	0	193	0	100
No regulated gene	135	50	245	92
No TFBS (at least 1)	154	58	367	131
No TFBS (all)	57	30	166	26
Conserved links	271	258	712	276
Total links	560	559	1324	599

## Discussion

Here we approached the evolution of regulatory interactions in the Enterobacteriales from three different angles.

The abundance of motifs in biological networks leads to a question of the evolution action on their edges. Previously, the main criterion of link persistence has been conservation of the TFs and the regulated gene [27,28,37,60,61]. But the fate of the third element, the TFBS, has not been taken into account, though TFBSes are the most plastic part of the network (one mutation in a DNA sequence is often sufficient to create a new or break an existing TFBS). So, they are the main instrument of incorporating or destructing interactions in the network, thus, the main engine of evolution. Here, we considered the transcriptional link as a set of three elements and studied the conservation of all three.

The set of genes involved in FFLs is enriched in COG categories “energy production” (p-value 0) and “carbohydrate transport and metabolism” (p-value 7,3x10^-7^). Indeed, most FFLs are formed by the global TFs Fnr and ArcA so that FNR regulates the *arcA* gene and both co-regulate genes from the former category; or the global regulator CRP and local regulators of carbohydrate catabolism operons [27]. The same trend was obtained using GeneOntology categories (data not shown). At that, it should be noted that the FNR-ArcA regulatory cascade itself is not conserved outside Enterobacteriales, as the relationships between these genes vary in three families of gamma-proteobacteria, Enterobacteriaceae, Pasteurellaceae and Vibrionaceae [30].

A naïve hypothesis that the network motifs are frequent because they are functionally important and hence more conserved turned out to be not entirely true. Our analysis demonstrates that the regulatory-link evolution depends on the link’s status in the TRN. In *E. coli* strains, local regulatory links indeed tend to be more conserved in FFLs. On the level of Enterobacteriales, the links persist better in non-motif connections. One possible explanation of the contradiction is that the Enterobacteriales transcriptional network is incomplete. We still do not know all TFs, regulated genes and TFBSs. Further, TFBSes may be too weak for the comparative computational analysis. On the other hand, the observed TRN properties at the level of *E. coli* strains seem to be robust, the fraction of conserved links stabilizes at some point and does not change after adding more strains.

Previous research also pointed to the importance of the motif usage: if an edge is not useful, it will be rapidly destroyed in evolution [62]. The local regulatory link in the coherent type C1 FFL turns out to be lost more often than in the incoherent type I1 FFL. As the transcription expression is regulated by two different regulators in the same direction, one of them might be considered as redundant and hence dispensable. In contrast, in the incoherent FFL, the expression is regulated in different directions, and in this case the loss of a regulatory link would destroy the whole expression mode.

We also developed a simple method for studying positively selected nucleotides in non-coding DNA based on the comparison of multiple strains in two related species. Hence it is complementary to the technique used by Molina and van Nimwegen [44] for quantifying evidence of purifying selection at noncoding positions in bacteria. They built explicit models of the substitution rates for each multiple-alignment column and calculated the likelihood-ratio *R* of the “background” and “foreground” model as an estimation of evidence that position is under purifying selection. The difference between models is that in “background” model for all positions substitutions from nucleotide β to nucleotide α are assumed to go at the same rate r_αβ_, while in “foreground” model the substitution rates are altered due to selection preferences for certain nucleotides at this position. Our method requires an assumptions likely satisfied in the performed analysis – the set of strains should be sufficiently diverse to obtain an unbiased set of polymorphisms. While the degree of strain relatedness in our sample is uneven, both samples contain numerous divergent strains. Moreover, since the method does not rely on allele frequencies in polymorphic sites, but only on the presence of polymorphisms, the presence of close strains does not pose a problem. Finally, the resampling procedure demonstrates the robustness of obtained estimates.

Molina and van Nimwegen calculated the distribution of *R* for different classes of positions in *E. coli*. Since no fixed threshold for *R* was established to unambiguously determine positions subject to purifying selection, direct comparison with our results is not possible. However, if we set threshold *R*=1.5 as a stringent criterion to discriminate between sites under selection and neutral positions, then rough estimates of the fraction of positions subject to purifying selection would coincide with our results: synvergons and divergons contain ~50% sites under selection while convergons contain ~15% of sites under selection. Thus Molina and van Nimwegen’s observation that upstream regions shows increased purifying selection compared to downstream regions is in agreement with our findings.

The calculated values allow one to estimate the fraction of positions in yet unknown functional sites. Indeed, if *L*_*xy*_ is the total length of known sites of type *x* (promoters, terminators, TFBSs) in regions of type *y* (divergons, convergons, synvergons), *L*_*y*_ is the total length of such regions, and ω_*x*_ and ω_*y*_ are the fractions of positions under selection in known sites of type *x* and regions of type *y*, respectively, then the total number of position under selection in the region of type *y* is *L*_*y*_ω_*y*_, whereas the number of position under selection in the known sites is Σ_*x*_(*L*_*xy*_ω_*x*_). Hence, the fraction of unknown positions is (*L*_*y*_ω_*y*_ – Σ_*x*_(*L*_*xy*_ω_*x*_))/*L*_*y*_ = ω_*y*_ – Σ_*x*_(*L*_*xy*_ω_*x*_)/*L*_*y*_. This calculation yields 44%, 35%, and 8% unknown, selected positions in the synvergons, divergons and convergons, respectively. Said in another way, known sites explain 14%, 24%, and 24% of all positions under negative selection in the synvergons, divergons and convergons, respectively. We recalculated our estimates after excluding intergenic regions containing RNA-based regulatory structures such as riboswitches and attenuators as well as genes that encode small RNAs, and observed small, statistically insignificant differences in the estimated fraction of positions subject to purifying selection (data not shown).

Positions variable at different clades could arise from ancient polymorphisms. While this does not affect our calculations and conclusions, as positions polymorphic in the last common ancestor of *E. coli* and *Salmonella* and retaining this polymorphism are likely neutral, it is of interest to compare the allelic content of different types of positions (Table 4). At that, the fraction of situations where two lineages have different alleles is roughly 20% both in monoallelic positions (of the *CC* and *CD* types) and in positions where one lineage is constant and the other linage biallelic (the *CN* and *NC* types).

**Table 4:** Distribution of allelic variants

	**1 allele in** ***Salmonella***	**2 alleles in** ***Salmonella***	**3 alleles in** ***Salmonella***	**4 alleles in** ***Salmonella***
1 allele in *E.coli*	17541 + 3824	1087 + 245	33 + 4	0
2 alleles in *E.coli*	594 + 126	153	7	0
3 alleles in *E.coli*	24 + 4	9	1	0
4 alleles in *E.coli*	1	0	0	0

The first number in cells from the first row and the first column reflects cases where the single allelic variant in one lineage is among the allelic variants from the other lineage, and the second number reflects the cases where the allelic variants are different. The mean values for all parameters were calculated for 100 bootstrap samples of 15 *E. coli* strains and 15 *Salmonella* strains.

Direct comparison of levels of purifying selection in non-coding regions between eukaryotic and prokaryotic genomes is complicated by the fact that the fraction of non-coding DNA in bacterial genomes is 6-14%, while eukaryotic genomes have much more non-coding DNA. The smallest nuclear genome contains 22% intergenic DNA [63] and the single-celled eukaryotic model organism *S. cerevisiae* contains 30% intergenic DNA [64]. In genomes of multicellular eukaryotes, the fraction of noncoding DNA is close to 90% [65] with the intron length and number highly variable even among related species.

The fraction of functionally constrained intergenic regions in *S. cerevisiae* was estimated to be ~43% based on calculating the ratio of intergenic to synonymous substitution rate [42]. In the genome of protist *Theilleria parva*, ~35% of orthologous intergenic regions and ~30% of intronic regions are constrained [66]. In the genome of *D. melanogaster*, a substantial fraction (40-50%) of intronic and intergenic DNA seems to be under selection according to comparison with 4-fold degenerate (synonymous) sites in coding sequences [67,68]. Thus one can see that despite the differences in the fraction of non-coding DNA between bacteria and eukaryotes, various estimates give approximately 40% of non-coding sites subject to purifying selection.

Here we analysed only one bacterial group, Enterobacteriales, in which the genomes of a sufficient number of strains and closely related species were sequenced, and experimental data on transcriptional regulation were available. We plan to apply the developed methods not requiring experimental data to the analysis of other large groups with many sequenced member, in particular *Streptococcus* and *Burkholderis* spp.

## Conclusions

Overall, we have demonstrated that the naïve hypothesis that FFLs would be highly conserved turned out to be not entirely true. The conservation of regulatory interactions depends on their status in the transcriptional network, that is, whether they are involved in a FFL, is the FFL coherent or incoherent, is the regulator global or local. On the other hand, the developed simple method for estimating the strength of the negative selection in intergenic region provides results largely consistent with the observation made in other genomes. Advances in experimental and computational techniques of high-throughput data collection as well as sequencing of more genomes and hence increasing statistical power of comparative analyses will lead to reconstruction of more complete transcriptional regulatory networks. It will also show, whether the observed trends and estimates are universal for all bacteria.

## Methods

Complete bacterial genomes were obtained from GenBank [1] (Additional file 2: Table S2). Selection in intergenic regions was studied in 32 strains of *E. coli* (including *Shigella*) and 16 strains of *Salmonella enterica*. The evolution of regulatory interaction in network motifs was studied in 25 *E. coli* strains and 19 genomes of the *Enterobacteriales*.

Multiple alignments of intergenic regions were built using MUSCLE [69]. We generated 100 bootstrap samples of 15 *E. coli* strains and 15 *Salmonella* strains. For the analysis of selection, only intergenic regions between pairs of orthologous genes retaining the orientation in all 30 bootstrap strains were considered, whereas the analysis of site conservation in *E. coli* strains used regions upstream of orthologous genes that occur in more than ten strains.

Experimentally validated TF-binding sites, promoters and terminators of *E. coli* were obtained from the RegulonDB database [38]. FFLs members were extracted using ad hoc Perl scripts.

Transcriptional regulatory links were reconstructed in several steps. First, we identified orthologs of TFs and regulated genes. Orthologs were identified based on the bidirectional best-hit criterion [70] using ad hoc software developed with Oracle Express Edition (Oracle). The next step was the TFBSes reconstruction. For the *E. coli* strains, we analyzed multiple alignments of TFBSes in the intergenic regions. For the Enterobacteriales, positional weight matrices were either built using the Genome Explorer and SignalX programs based on TFBSes in RegulonDB [71] or obtained from the RegPrecise database [72]. If the TFBS weight decreased by more than 1 (in the SD units), we counted it as TFBS loss. We estimated the conservation of the regulatory links counting the number of those that remain unchanged in terms of TFs, TFBSs and regulated genes conservation. Statistical significance was assessed using the chi-squared test and the functional enrichment statistics was calculated using the hypergeometric distribution implemented in the R package.

## Competing interests

The authors declare that they have no competing interests.

## Authors’ contribution

MSG conceived and coordinated the project. MAP developed the method of estimating the fraction of positions under selection. OVT analysed the motif evolution. MDK constructed orthologous groups. MSG, MAP and OVT wrote the manuscript. All authors read and approved the final manuscript.

## Supplementary Material

Additional file 1 ** Table S1.** The number of regulated operons for *E. coli* transcription factors (Gama-Castro et al. 2011). Click here for file

Additional file 2 ** Table S2.** The list of studied genomes. A – analysis on the level of strains, B – analysis on the level of *E.coli* and closely related species, C – analysis on the level of closely related Enterobacteriales. Click here for file
